# Generation of human antibody fragments recognizing distinct epitopes of the nucleocapsid (N) SARS-CoV protein using a phage display approach

**DOI:** 10.1186/1471-2334-5-73

**Published:** 2005-09-19

**Authors:** Michela Flego, Paola Di Bonito, Alessandro Ascione, Silvia Zamboni, Alessandra Carattoli, Felicia Grasso, Antonio Cassone, Maurizio Cianfriglia

**Affiliations:** 1 Department of Drug Research and Evaluation, Istituto Superiore di Sanità, Rome, Italy; 2 Department of Infectious Parasitic and Immune-mediated Diseases, Istituto Superiore di Sanità, Rome, Italy

## Abstract

**Background:**

Severe acute respiratory syndrome (SARS)-CoV is a newly emerging virus that causes SARS with high mortality rate in infected people. Successful control of the global SARS epidemic will require rapid and sensitive diagnostic tests to monitor its spread, as well as, the development of vaccines and new antiviral compounds including neutralizing antibodies that effectively prevent or treat this disease.

**Methods:**

The human synthetic single-chain fragment variable (scFv) ETH-2 phage antibody library was used for the isolation of scFvs against the nucleocapsid (N) protein of SARS-CoV using a bio panning-based strategy. The selected scFvs were characterized under genetics-molecular aspects and for SARS-CoV N protein detection in ELISA, western blotting and immunocytochemistry.

**Results:**

Human scFv antibodies to N protein of SARS-CoV can be easily isolated by selecting the ETH-2 phage library on immunotubes coated with antigen. These *in vitro *selected human scFvs specifically recognize in ELISA and western blotting studies distinct epitopes in N protein domains and detect in immunohistochemistry investigations SARS-CoV particles in infected Vero cells.

**Conclusion:**

The human scFv antibodies isolated and described in this study represent useful reagents for rapid detection of N SARS-CoV protein and SARS virus particles in infected target cells.

## Background

The widespread diffusion and mortality of severe acute respiratory syndrome (SARS), caused by a new *Coronavirus *(SARS-CoV) has threatened the entire world [1] and has urged the scientific community to develop molecular and serological tests which can assist for rapid detection of SARS cases and implementation of control measures [2]. Effective prophylaxis and antiviral therapies are urgently needed in the event of re-emergence of the highly contagious and often fatal SARS infection.

Like other known *Coronaviruses*, SARS-CoV is an enveloped virus containing four structural proteins, namely the membrane (M), envelope (E), spike (S) and the nucleocapsid (N) proteins [1]. The N protein is a 423 amino acid, predicted phospho-protein of 46 kDa, which shares little homology with other members of the *Coronavirus *genus [3,4]. Several studies have found that N protein is highly immunogenic, thus antibody response in patients with SARS is directed most frequently and predominantly to the nucleocapsid. It has also been found that anti N antibodies are detected early and with high specificity during infection [5]. Therefore, the generation of N-specific reagents, in particular monoclonal antibodies (mAbs) could be of assistance in studies of viral replication and antiviral activity, as well as in the diagnosis of SARS-CoV infection at various disease stages. It has also been found that SARS patients show clinical improvement if they are treated with serum from previously infected patients suggesting that passive immunotherapy could be developed for the treatment of this infectious disease [6]. However, the traditional approach to generate mAbs to SARS-CoV has presented difficulties for several reasons including safety concerns in handling SARS particles [7]. To overcome these limitations, we applied a very effective and safe in *in vitro *procedure based on human scFvs selection from the large synthetic ETH-2 phage antibody library [8]. Here, we report the identification, production, and epitope characterization of human scFv antibodies specifically recognizing distinct N protein domains. These recombinant mAbs were found to bind selectively and with good affinity to distinct N protein epitopes, and were suitable to specifically identify SARS particles in Vero infected cells.

## Methods

### SARS-CoV antigens

Nucleocapsid (N) protein of SARS-CoV (residues: 1–422) and its fragments N1 (residues: 1–117), N2 (residues: 110–340) and N3 (residues: 333–422) expressed in *E. coli *by DNA recombinant technology as reported by Carattoli, Di Bonito et al [9]. These polypeptides have proved to react with specific sera of SARS-CoV infected patients.

### ETH-2 antibody phage library

The synthetic human recombinant antibodies library (ETH-2) consists of a large array (more than 10^9 ^antibody combination) of scFv polypeptides displayed on the surface of M13 phage. It was built by random mutagenesis of the CDR3 of only three antibody germline gene segments (DP47 for the heavy chain, DPK22 and DPL16 for the light chain). Diversity of the heavy chain was created by randomizing four to six positions replacing the pre-existing positions 95 to 98 of the CDR3. The diversity of the light chain was created by randomizing six positions (91 to 96) in the CDR3 [8].

### Isolation of phage antibodies from ETH-2 library

An aliquot of the ETH-2 library, containing 10^13 ^cfu phage, was used to isolate specific human antibodies in scFv format to SARS-CoV N protein. Immunotubes (Nunc Maxisorp; Denmark) were coated overnight (ON) at room temperature (RT) with 10 μg/ml of recombinant purified SARS-CoV N protein in PBS. After panning, phages were eluted with 1 ml of 100 mM triethylamine and the solution was immediately neutralized by adding 0.5 ml of 1 M Tris-HCl pH 7.4. Eluted phages were used to infect log phase TG1 *E. coli *bacteria and amplified for the next round of selection. Briefly, 50 ml of 2 × YT with 100 μg/ml ampicillin (2 × YTA medium) and glucose 1% were inoculated with enough bacterial suspension to yield an OD_600 nm_≅ 0.05–0.1. The culture was grown up to OD_600 nm _0.4–0.5 and infected with M13 K07 helper phage in a ratio of around 20:1 phage/bacteria. The rescued phages were concentrated by precipitation with PEG 6000 and used for next round of panning (usually three to recover antigen-specific antibody phages from the ETH-2 library). For soluble scFv preparation, individual colonies were grown in 96 flat bottomed wells (Nunc) for 2 hours at 37°C in 180 μl 2 × YTA medium and glucose 0.1% in 96 well plates and inducted with 50 μl 2 × YTA medium and 6 mM IPTG. The following day the plates were spinned down at 1800 g for 10 minutes and the supernatants containing soluble scFvs were recovered and tested for specificity.

### ELISA

96 well ELISA-plates (Nunc Maxisorp) were coated ON at RT with 0.5 μg of antigen (N protein or its N1, N2, N3 fragments) in PBS. The following day a blocking solution, consisting of 2% non fat dry milk in PBS (MPBS), was added; plates were washed with PBS containing 0.05% Tween 20 (TPBS) and incubated for 2 hours at RT with 50 μl of supernatants containing soluble scFv antibody, anti-Flag M2 antibody (1.6 μg/ml Sigma-Aldrich; MO, USA) and anti-mouse HRP conjugated antibody (1.6 μg/ml Dako; Denmark). The reaction was developed using 3, 3^1^-5, 5^1^-tetramethylbenzidin BM blue, POD-substrate soluble (Roche Diagnostics; IN, USA) and stopped by adding 50 μl of 1 M sulfidric acid. The reaction was detected with an ELISA reader (Biorad; CA, USA), and the results were expressed as A (absorbance) = A(450 nm)-A(620 nm).

### Western blotting

1 μg of SARS-CoV N, N1, N2 and N3 proteins were loaded onto 12% SDS-PAGE and transferred to a nitrocellulose membrane using standard procedures. The membrane was blocked in 4% MPBS ON at RT. Blotted proteins were incubated for 2 hours with supernatant containing soluble scFvs, washed with 0,05% TPBS and incubated again with 5 μg/ml anti-Flag M2 mouse antibody (Sigma-Aldrich) in 2% MPBS. After an additional incubation for 1 hour at RT in presence of 5 μg/ml of a goat anti-mouse antibody HRP-conjugated (Dako), the reaction was developed and visualized with a chemiluminescence detection kit (Pierce; IL, USA).

### Immunocytochemical determination of SARS-CoV particles

BIOCHIP slides (Euroimmun, Luebeck, Germany) coated with SARS-CoV infected Vero cells were used for immunocytochemistry determination of virus particles by phage or scFv specific antibodies. Slides were treated for 10 minutes with peroxidase block solution (Dako En Vision System HRP), washed in TPBS buffer and incubated for 1 hour at RT with 10^12 ^phage antibody particles (resuspended in 25 μl of TPBS) or scFv containing supernatant (25 μl). As control, BIOCHIP slides were incubated with an irrelevant anti-glucose oxidase phage or scFv antibodies [10]. After extensive washings with TPBS, the slides were incubated for 1 hour at RT with an anti-M13 secondary mouse antibody (5 μg/ml in 20 μl TPBS) (Amersham) or with an anti-FLAG secondary mouse antibody (5 μg/ml in 20 μl TPBS) (Sigma-Aldrich) for labelling phage and scFv reactive antibodies, respectively. After washings, the slides were treated for 30 minutes at RT with cromogen solutions (Dako) and inspected with a light microscope.

### DNA characterization and sequences

Plasmid DNA from individual bacterial colonies of MA2.D5, MA2.D7 and MA2.E3 clones was digested with specific endonucleases and CDR3 regions sequenced with an automated DNA sequencer (M-Medical/Genenco, Pomezia, Italy) using fdseq1 (5'-GAA TTT TCT GTA TGA GG-3') and pelBback (5'-AGC CGC TGG ATT GTT ATT AC-3') primers.

## Results and discussion

### Isolation of human scFv antibodies

**Figure 1:**
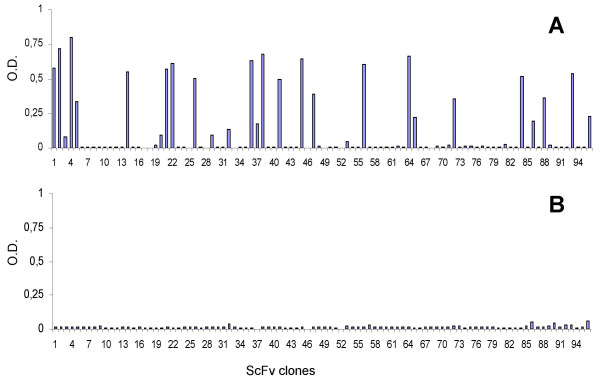
**N protein specific scFvs antibodies determined by ELISA. **IPTG inducted bacterial supernatants of individual colonies from the third round of the ETH-2 library selection on N protein were tested in 96-well microtiter plates coated with antigen or glucose oxidase (negative control). ELISA readings higher than three fold above negative controls were scored as positive reactions. ELISA values of the scFv clones against N protein (panel A) and glucose oxidase (panel B) are shown.

To isolate specific scFv antibodies, an aliquot of the human synthetic ETH-2 library containing approximately 1 × 10^12 ^cfu phages was introduced for panning into Maxisorp immunotubes coated with purified SARS-CoV N protein as antigen. Non-specifically absorbed phages were removed by washing. Bound phages were eluted, amplified and used for the next cycle of panning as described elsewhere [8,10]. By using this protocol [10], phage antibody populations specifically recognizing the N protein of SARS-CoV were isolated after only three rounds of selection. Plating on agar of TG1 phage antibody-infected cells, allowed growth of individual phagemid clones. Soluble scFvs derived from IPTG inducted colonies, were screened by ELISA and several of them proved to be specific for N protein (Figure 1).

### Epitope recognition

**Figure 2:**
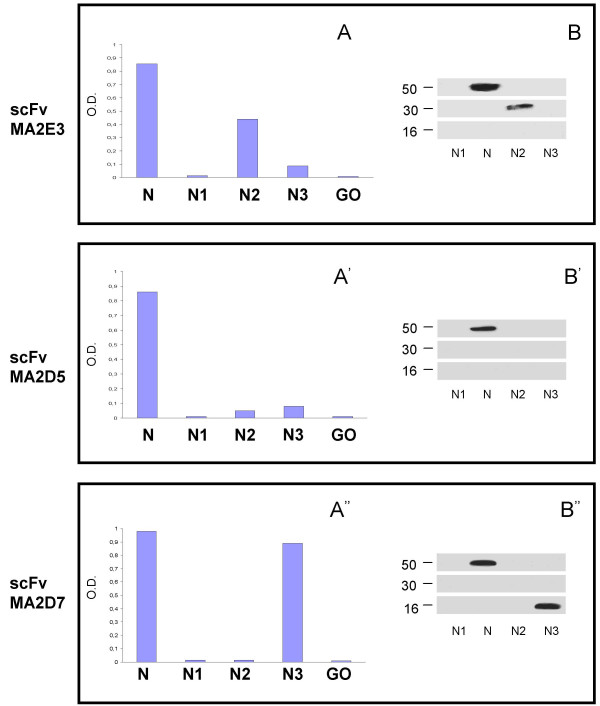
**N protein domain recognition by specific scFv antibodies. **The scFvs MA2.E3, MA2.D5 and MA2.D7 antibody clones showing distinct N protein recognition patterns in an ELISA (A, A', A") were also analyzed for different epitope recognition by western blot. While all scFv antibodies react with a 46–48 kDa band corresponding to the MW of the N protein (B, B', B"), the scFvs MA2.E3 and MA2.D7 also react with a 28–30 KDa and 12–14 KDa band (B, B") corresponding to the MW of N2 and N3 protein fragments, respectively.

N-positive scFv clones were analysed by ELISA for reactivity with N1, N2 and N3 protein fragments (Figure 2). Three distinct classes of scFv antibodies recognizing either the intact N protein only or also N2 or N3 fragments were identified. ScFv antibodies recognizing N protein only but none of its fragments (for instance the MA2D5 scFv) were likely directed against epitopes encompassing two adjacent protein fragments or conformational epitopes expressed only on the integral protein. The absence of scFv antibodies reacting with the N1 fragment somewhat matches the lower antigenicity of this polypeptide, as compared to immunodominant N2 and N3 fragments, [9], despite the reported reactivity of several linear synthetic epitopes of the N1 region with SARS sera [11]. However, a relatively low number of scFv clones were here tested for a sound conclusion on this aspect.

### SARS-CoV particles identification

Three of the most reactive scFv antibody clones named MA2.D5, MA2.D7 and MA2.E3 each one representing the different classes of epitope recognition in the N protein domains were further assessed to verify their biochemical patterns, reactivity with SARS-CoV infected cells, and DNA sequences of their encoding genes.

Western blotting analysis (Figure 2) shows that all three antibodies react with a 46 kDa band corresponding to the predicted molecular mass of the intact N protein [3]. As expected by previous ELISA indications, the scFvs MA2.E3 and MA2.D7 recognize also a 28–30 kDa and 12–14 kDa bands respectively, corresponding to the predicted molecular mass of N protein fragments N2 and N3 [9].

**Figure 3:**
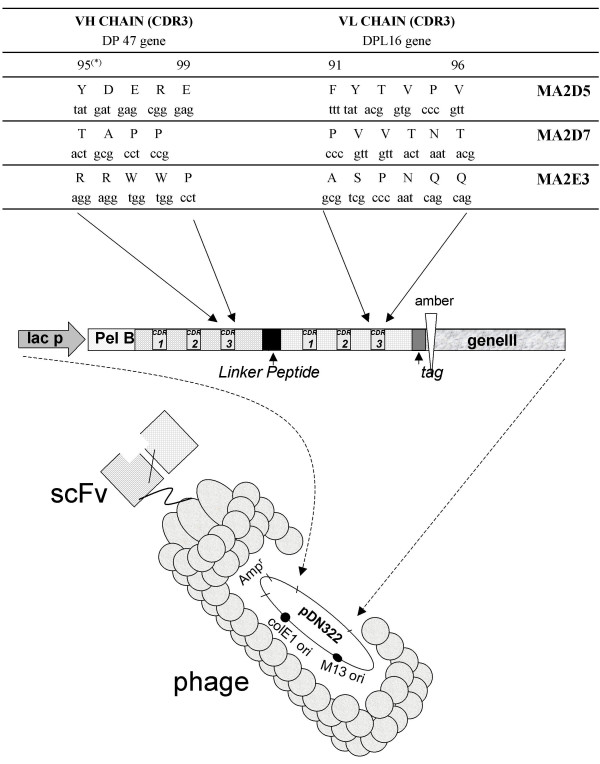
**Molecular genetics characteristics of the scFv antibodies. **The nucleotide composition, and the corresponding amino acid sequences and residue position in the CDR3 region of the selected scFv antibodies MA2.D5, MA2.D7 and MA2.E5 are reported. A schematic representation of the scFv antibodies displayed on M13 phage as pIII fusion proteins is depicted.

**Figure 4:**
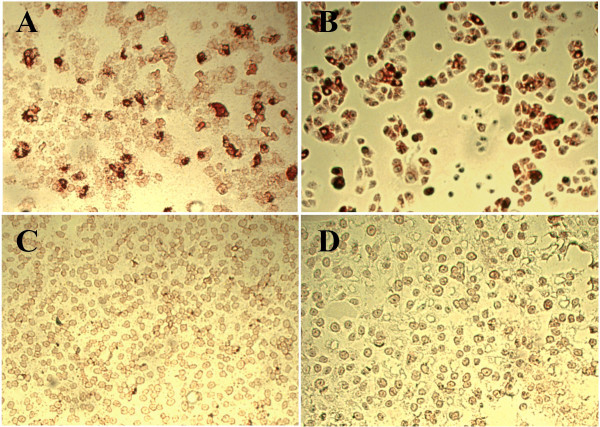
**SARS-CoV detection by MA2.D5 scFv antibody. **The immunocytochemical detection of SARS-CoV particles in infected monkey Vero cells by the MA2.D5 antibody is shown in A (soluble scFv protein) and B (phage displaying scFv antibody). In C and D the reactivity of Vero cells with an irrelevant scFv and phage antibody are shown.

The molecular analysis of the phage antibodies MA2.D5, MA2.D7 and MA2.E3 shows scFv antibodies with a molecular weight of about 27 kDa and the integrity of the genes encoding for the cognate scFvs displayed on M13 phage (data not show). VH and VL gene sequences, shows that each single scFv antibody clone possess a unique DNA sequence encoding for the CDR3 region (Figure 3); this uniqueness is consistent with the distinct epitope recognition patterns of the three scFvs noted above. Immunocytochemical investigations show that MA2.D5 antibody specifically recognizes SARS-CoV particles in infected Vero cells (Figure 4). The other two antibodies MA2.D7 and MA2.E3, though genuinely reacting with N protein and its N2 and N3 fragments in ELISA and western blotting (see above) did not prove to be useful for SARS-CoV determination in infected cells.

## Conclusion

The usefulness of the library as a tool for generating monoclonal antibodies against viral pathogens [12-14] including SARS-CoV [15,16] has been tested and showing that phage antibodies may recognize viral proteins used as antigens. In particular, a rodent library has proved to be very effective for the isolation of murine scFvs exhibiting high specificity against SARS-CoV E protein, which is recognized as a 31 kDa protein in western blot studies [15]. Interestingly, one of these clones (A17) isolated by selecting the rodent library against the 31 kDa E protein cross-reacts in ELISA with a well-defined fragment of the 46–48 kDa N protein. Recently, human mAbs were identified by biopanning of a synthetic library on SARS-CoV lysate; the isolated scFvs expressed on phages have proved to recognize N protein only in ELISA while no evidences for N protein recognition in western blot studies or in SARS infected cells were shown [16]. The efficient display of recombinant antibodies on filamentous phage has been also assessed by our findings showing that soluble human scFvs selected from the ETH-2 library may yield agents capable of specifically recognizing SARS-CoV particles in infected Vero cells as well as the intact 46–48 kDa SARS-CoV N protein and its N2 (28–30 kDa) and N3 (12–14 kDa) fragments in western blot and ELISA investigations (Figure 2 and 4). To our knowledge, the human scFvs directed against N protein, by us isolated and characterized, are the first antibodies so far described which are able to detect SARS-CoV N protein in different routinary laboratory techniques such as ELISA, Western blot and immunocytochemistry. Hence, these human mAbs represent excellent candidates for future development of serological diagnosis of SARS-CoV infections since high functionality (all clones express soluble antibodies in bacteria), specificity (all clones recognize the 46–48 kDa N protein) and quality (all clones give good signals in ELISA, in western blot as well and stain SARS-CoV infected cells).

Furthermore, the genes encoding for the selected MA2.D5, MA2.D7 and MA2.E3 antibodies have been isolated and sequenced, thus facilitating various molecular approaches including site direct mutagenesis to maturate binding affinity and construct whole recombinant immunoglobulins for SARS-CoV studies and applications. To this latter regard, another immediate use of human scFv directed to N protein is through intracellular expression as a novel strategy of gene therapy aimed at knockout the replicative cycle of SARS-CoV in infected cells. It is thought that intracellular expression of scFv antibodies may be superior over alternative methods for gene inactivation such as anti-sense RNA and dominant negative mutants because of its high specificity [17,18].

Nevertheless, phage display approach make feasible several strategies for the isolation of neutralizing human mAbs to provide an immediate treatment for emergency prophylaxis of SARS-CoV [19] while vaccines and new drugs are underway. For example, immune library may be constructed and screened on SARS-CoV proteins, using B-cells from convalescent SARS patients as a genetic source of specific VH and VL. In alternative, synthetic peptides mimicking immunodominant epitopes in S or M SARS-CoV proteins [20] may be used as a substrate antigens for the identification of specific scFvs from synthetic human phage antibody library.

## List of abbreviations

SARS, Severe Acute Respiratory Syndrome; CoV, *Coronavirus*; N, Nucleocapsid; scFv, single chain fragment variable; ETH-2, synthetic scFv antibody phage library; CDR, complementarity determining regions; mAbs, monoclonal antibodies.

## Competing interests

The author(s) declare that they have not competing interest.

## Authors' contributions

MF carried out phage antibodies and scFvs selection from all different N-antigens, participated in the genetic molecular, biochemical and immunohistochemical characterization of scFvs specific for distinct epitopes of N protein.

PDB and FG carried out isolation, expression, production and purification of nucleocapsid protein from SARS-CoV.

AA participated in phage antibodies selection and carried out immunoassay and biochemical characterization of scFvs against N protein and its polypeptide fragments.

ACr firstly, ideated and developed a genetic molecular model to isolate, identify and express SARS-CoV N protein and its fragments.

AC promotes the genetic molecular study of SARS-CoV N protein, participated in the design of the study, supervisioned the experiments and critically revised the manuscript. MC conceived of the study, promotes the approach with phage library to select specific scFv human antibodies against SARS-CoV protein. Furthermore, MC participated in the design and coordination of the research and drafting the manuscript.

All authors have read and approved the final version of the manuscript.

## Pre-publication history

The pre-publication history for this paper can be accessed here:


